# Retracted: A Newborn with Genital Ambiguity, 45,X/46,XY Mosaicism, a Jumping Chromosome Y, and Congenital Adrenal Hyperplasia

**DOI:** 10.1155/2022/9808430

**Published:** 2022-02-22

**Authors:** Case Reports in Endocrinology

At the request of the authors, the article titled “A Newborn with Genital Ambiguity, 45,X/46,XY Mosaicism, a Jumping Chromosome Y, and Congenital Adrenal Hyperplasia” [1] has been retracted. The article stated that “Full written consent has been obtained from the parents of the patient for the publication of this paper.” However, the parents have since stated that they did not understand the full scope of this disclosure and have now withdrawn their consent.
